# The role of insulin receptor substrate 1 gene polymorphism Gly972Arg as a risk factor for ischemic stroke among Indonesian subjects

**DOI:** 10.1186/s13104-018-3823-6

**Published:** 2018-10-11

**Authors:** Syahrul, Samekto Wibowo, Sofia Mubarika Haryana, Indwiani Astuti, Fariz Nurwidya

**Affiliations:** 1 Department of Neurology, Faculty of Medicine, Universitas Syiah Kuala, Banda Aceh, Indonesia; 2 Department of Neurology, Faculty of Medicine, Universitas Gadjah Mada, Yogyakarta, Indonesia; 3 Department of Molecular Biology, Faculty of Medicine, Universitas Gadjah Mada, Yogyakarta, Indonesia; 4 Department of Pulmonology and Respiratory Medicine, Faculty of Medicine, Universitas Indonesia, Persahabatan Hospital, Jalan Persahabatan Raya No.1, Rawamangun Jakarta, 13230 Indonesia; 5 Department of Nutrition, Faculty of Medicine, Universitas Indonesia, Dr. Cipto Mangunkusumo Hospital, Jakarta, Indonesia

**Keywords:** Ischemic stroke, Metabolic syndrome, Insulin resistance, IRS-1 gene polymorphism Gly972Arg

## Abstract

**Objective:**

The identification of new genetic-associated risk factor of ischemic stroke could improves strategies for stroke prevention. This study aims to identify insulin receptor substrate 1 (IRS-1) gene polymorphism Gly972Arg as the risk factor for ischemic stroke among Indonesian subjects. The case–control study was conducted by matching the gender and race on 85 cases of patients with ischemic stroke and 86 healthy non-stroke control subjects. Ischemic stroke was established by the complete neurology examination and brain computed tomography scan or magnetic resonance imaging. Polymerase chain reaction–Restriction Fragment Length Polymorphism was performed to analyze IRS-1 gene Gly972Arg genotype.

**Results:**

There were 85 ischemic stroke cases and 86 control subjects. The distribution of nucleotide IRS-1 gene polymorphism Gly972Arg in the ischemic stroke vs health controls for GG were 32.2% vs 41.5%, for GR were 16% vs 7.6%, and for RR were 0.5% vs 1.9%. IRS-1 gene polymorphism Gly972Arg was found as significant risk factor for ischemic stroke [odds ratio of 2.6 (1.27–5.27); CI 95%, *p *= 0.008]. Conclusively, the IRS-1 gene polymorphism Gly972Arg should be considered as an important factor in the prevention and treatment of ischemic stroke.

**Electronic supplementary material:**

The online version of this article (10.1186/s13104-018-3823-6) contains supplementary material, which is available to authorized users.

## Introduction

Substantial genetic component defects exist in ischemic stroke. The identification of new genetic-related risk factor for ischemic stroke is needed to improve stroke prevention strategies and determine the potential target of the ischemic stroke treatment.

Hypertension, diabetes mellitus, heart disease, history of TIA (transient ischemic attack) and stroke history have long been recognized as major risk factors of stroke [1, 2]. Furthermore, metabolic syndrome and insulin resistance are closely related with the incidence of stroke [3–7].

The prevalence of the metabolic syndrome is high among men and women [8]. As an indicator of insulin resistance, the insulin receptor substrate 1 (IRS-1) has been associated to ischemic stroke [9]. The IRS-1 polymorphisms has been linked to high platelet reactivity in coronary artery disease (CAD) patients with type 2 diabetes mellitus (T2DM) [10, 11]. It has been reported that IRS-1 polymorphism genotype Gln192Arg is associated with the increased incidence of ischemic stroke [12]. Moreover, phosphodiesterase 4D (PDE4D) [13], and 5-lipoxygenase gene activating protein (ALOX5AP) [14], were shown to be significantly related with ischemic stroke due to progressive changes in the walls of blood vessels resulting in the increased atherosclerosis process. These studies suggest that human gene variation that contributed to the pathogenesis of stroke is crucial to be considered in the prevention and treatment of the disease [15].

Metabolic syndrome is consists of abdominal obesity, hyperglycemia, insulin resistance, hypertriglyceridemia, low HDL cholesterol and hypertension and is an important risk factor for atherosclerotic cardiovascular disease [16]. The most commonly detected polymorphism in IRS-1 is a glycine to arginine change at codon 972 (Gly972Arg) [17]. Previous study found that metabolic syndrome is related with IRS-1 gene polymorphism Gly972Arg [18].

The current study addresses the question on whether IRS-1 gene polymorphism Gly972Arg acts as the risk factor for ischemic stroke among Indonesian subjects.

## Main text

### Materials and methods

This is a case control study by matching the gender and race of cases of ischemic stroke with healthy non-stroke control subjects. The inclusion criteria for ischemic stroke patients was acute ischemic stroke cases with onset of stroke within 7 days, aged 35–75 year old, Malay race, and treated in the Dr. Zainoel Abidin Hospital, Banda Aceh, Indonesia, and provided written consent to be enrolled in the study. The controls were healthy participants not suffering from stroke, without history of stroke and without family history of stroke who were willing to follow the study by signing the informed consent.

Subjects underwent the following procedures: general physical examination; complete neurological examination; non-contrast brain computed tomography (CT) scan or non-contrast magnetic resonance imaging (MRI) to confirm ischemic stroke. The CT scanner system used was SOMATOM Sensation 64-slice CT scanner and the MRI system used was 3T Magnetom (Siemens Healthcare Corp, Erlangen, Germany). Hypodense lesion from brain CT scan or hypointense lesion from MRI were considered as positive results for cerebral infarct, DNA isolation was performed and followed by Polymerase chain reaction–Restriction Fragment Length Polymorphism (PCR–RFLP) screening using the enzyme SmaI for genotype analysis of IRS-1 gene Gly972Arg. The forward primer 5′CTTCTGTCAGGTGTCCATCC3′ forward and reverse primer 5′TGGCGAGGTGTCCACGTAGC3′ used in this study were designed by the Primer BLAST with NCBI Reference Sequence Number: NM_005544.2 (https://www.ncbi.nlm.nih.gov/nucleotide/187761322). Change of nucleotide GGG to become RGG is defined as IRS-1 gene polymorphism Gly972Arg. For the control subjects, the same procedures were performed except for the brain CT scan or MRI.

Data was subject to statistical analysis by using Statistical Package for the Social Sciences (SPSS) Windows software ver. 19.0 (Chicago, IL, USA). Bivariate categorical data was statistically analyzed by Chi squared test. Odds ratio (OR) with 95% of confidence interval (CI) was calculated to show association between variables. *P* < 0.05 was considered to be statistically significant.

### Results

This study involved 85 cases with ischemic stroke with the mean age of 57.3 years, and 86 control subjects with mean age of 44.2 years. The distribution of nucleotide IRS-1 gene polymorphism Gly972Arg in the ischemic stroke vs health controls for GG were 32.2% vs 41.5%, for GR were 16% vs 7.6%, and for RR were 0.5% vs 1.9%.

**Fig. 1:**
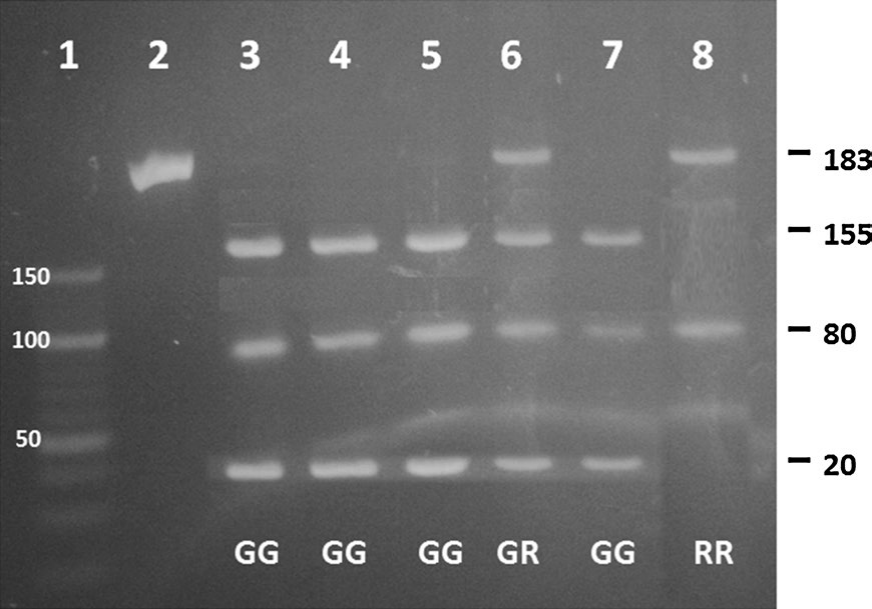
The result of the electrophoresis PCR–RFLP examination of IRS-1 Gene Polymorphism Gly972Arg. RFLP was conducted with *Sma*I restriction enzymes *Sma*1. Path 1: Marker ladder 50, Path 2: Control (+), Path 3, 4, 5, 7: GG Genotype, Path 6: GR Genotype, Path 8: RR Genotype

We performed the electrophoresis PCR–RFLP examination to determine the gene IRS-1 polymorphism Gly972Arg in some of the ischemic stroke cases as illustrated in Fig. 1. The length of DNA amplification was 263 base pairs (bp). By using the *Sma*I enzyme-mediated restriction, the DNA amplification would be cut into three bands called normal genotype (GG) consisting of the band of 155 bp band, 80 bp band and 28 bp band. Meanwhile, the genotype mutant heterozygote (GR) consisted of four bands: 183 bp; 155 bp; 80 bp; and; 28 bp. In addition, the genotype mutant heterozygote (RR) consisted of two bands: 183 bp, and; 80 bp.

Several representative subjects’ samples (subject number 9, 29, 46, and 48) were analyzed by sequence analysis to confirm the results of PCR–RFLP examination. We confirmed that PCR–RFLP results were consistent with the results of the IRS-1 gene sequence analysis (Additional file 1). Furthermore, the electrophoresis image for each PCR–RFLP results were considered to be accepted, namely the genotype sample number 9 (GG), 29 (GG), 46 (GG) and 48 (GR).

Next, we statistically analyzed the role of IRS-1 gene polymorphism Gly972Arg as the factor risk for ischemic stroke (Table 1). We found that IRS-1 gene polymorphism Gly972Arg was a significant risk factor for ischemic stroke.

**Table 1:** The role of IRS-1 gene polymorphism Gly972Arg as a risk factor for ischemic stroke

Variables	Mutant	Polymorphism		OR (CI 95%)	X^2^	*p*
		Mutant	Not mutant			
Samples	Case	30	55	2.6 (1.27–5.27)	7.02	0.008*
	Control	15	71			

*p* value is based on Chi-square test, *p < 0.05 was considered to be statistically significant

Finally, we determined the relationship between G972 and R972 alleles of gene IRS-1 with ischemic stroke as described in Table 2. The R972 allele (mutant) was significantly related to stroke ischemic (*p *= 0.008), whereas G972 allele (normal) showed no significant relationship.

**Table 2:** The R972 alleles of gene IRS-1 is significantly associated with ischemic stroke

Variables	IRS-1 gene G allele				IRS-1 gene R allele			
	Not allele G	Allele G	OR	*p*	Allele R	Not allele R	OR	*p*
Group								
Stroke	1	84	0.5 (0.04–5.6)	0.504	30	55	2.5 (1.2–5.3)	0.008*
Control	2	84			15	71		

*p* value is based on Chi-square test, *p < 0.05 was considered to be statistically significant

### Discussion

The investigation of potential detectable biomarker as risk factor in order to determine necessary primary prevention of stroke is crucial. This study shows that IRS-1 gene polymorphism Gly972Arg acts as the risk factor for ischemic stroke. Pathogenesis and molecular changes occurring in IRS-1 gene polymorphism Gly972Arg acting as the factor for ischemic stroke take place because of a mutation of the amino acid glycine by arginine at codon 972 substitution (G972R) [19]. This mutation resulted in the declined insulin sensitivity [20]. IRS-1 protein is expressed in the tissue and is very sensitive to insulin with a critical role in modulating the effect of insulin [21, 22]. Once attached to the insulin receptor, the specific tyrosine residues of IRS-1 protein will be phosphorylated which mediates insulin-controlled variety of cell functions, growth and metabolism [21, 23, 24]. Other study showed that IRS-1 gene Gly972Arg mutation has a strong relationship with the environment and genetic factor [25].

It has been previously reported that some of IRS-1 gene variants increases the risk of metabolic syndrome [26]. The pathogenesis of ischemic stroke due to metabolic disorders and insulin resistance is begun from changes at the cellular level with the glucose transporter type 4 (GLUT-4) translocation mechanism and the path of insulin transporter regulating the glucose metabolism within cells [27].

Botnia study showed around 10% of the population with normal glucose tolerance, 40% of glucose intolerance and 70% patients with DM-2 experiencing metabolic syndrome [28]. Metabolic syndrome increases the risk up to three times for heart coronary disease, myocardial infarction and stroke [29]. The insulin resistance underlies the pathological process of metabolic syndrome. Glucose intolerance and insulin resistance are estimated to cause hypertension, microalbuminuria, dyslipidemia and sympathetic hyperactivity of the nervous system [30].

IRS that phosphorylated in multiple residual tyrosines are the candidate of the genetic insulin resistance [31]. Polymorphism of two amino acids can be described in the IRS-1 gene that is insulin-sensitive and in the insulin-like growth factor 1 (IGF1) sensitive tissues. In one study, from 86 patients without relationship with NIDDM and 76 normoglycemic control, 10 subjects were shown to have NIDDM and three controls experience heterozygous polymorphism in codon 972 with glycine substituted by arginine [32].

Impaired IRS act as a mediator of signal transduction that contributed in the insulin resistance. IRS-1 phosphorylation on serine 307 occurred because of protein kinase/c-Jun N-terminal kinase (SAPK/JNK) activation as the critical part for the molecular change that leads to insulin resistance [33]. The inhibitor serine phosphorylase is the potential molecular mechanism of insulin resistance [34]. The combination of p85alpha and the increase in serine phosphorylation from IRS-1 will accelerate pathogenesis of insulin resistance [35].

To the best of our knowledge, this study is the first to report the role of IRS-1 gene polymorphism Gly972Arg as a significant risk factor for ischemic stroke in Indonesian subjects.

## Limitations

The nature of the case–control design in this study is not establishing cause and effect relationship. Although IRS-1 gene polymorphism Gly972Arg might serve as a predictor for ischemic stroke in the future, it is difficult to be considered as actionable target.

## Additional file

**Additional file 1.** The result of the sequence analysis of several sample research to identify IRS-1 gene polymorphism Gly972Arg. (A) Image A shows locust 972 where mutation G → R frequently occurs. (B) Image B shows locust 324 where in the sequence of amino acid group Lysine to Arginine mutation.
