# Supplementary figures and images for: The role of insulin receptor substrate 1 gene polymorphism Gly972Arg as a risk factor for ischemic stroke among Indonesian subjects
Source: BMC Res Notes. 2018 Oct 11;11:718. doi: 10.1186/s13104-018-3823-6 (PMC6180639; doi:10.1186/s13104-018-3823-6)

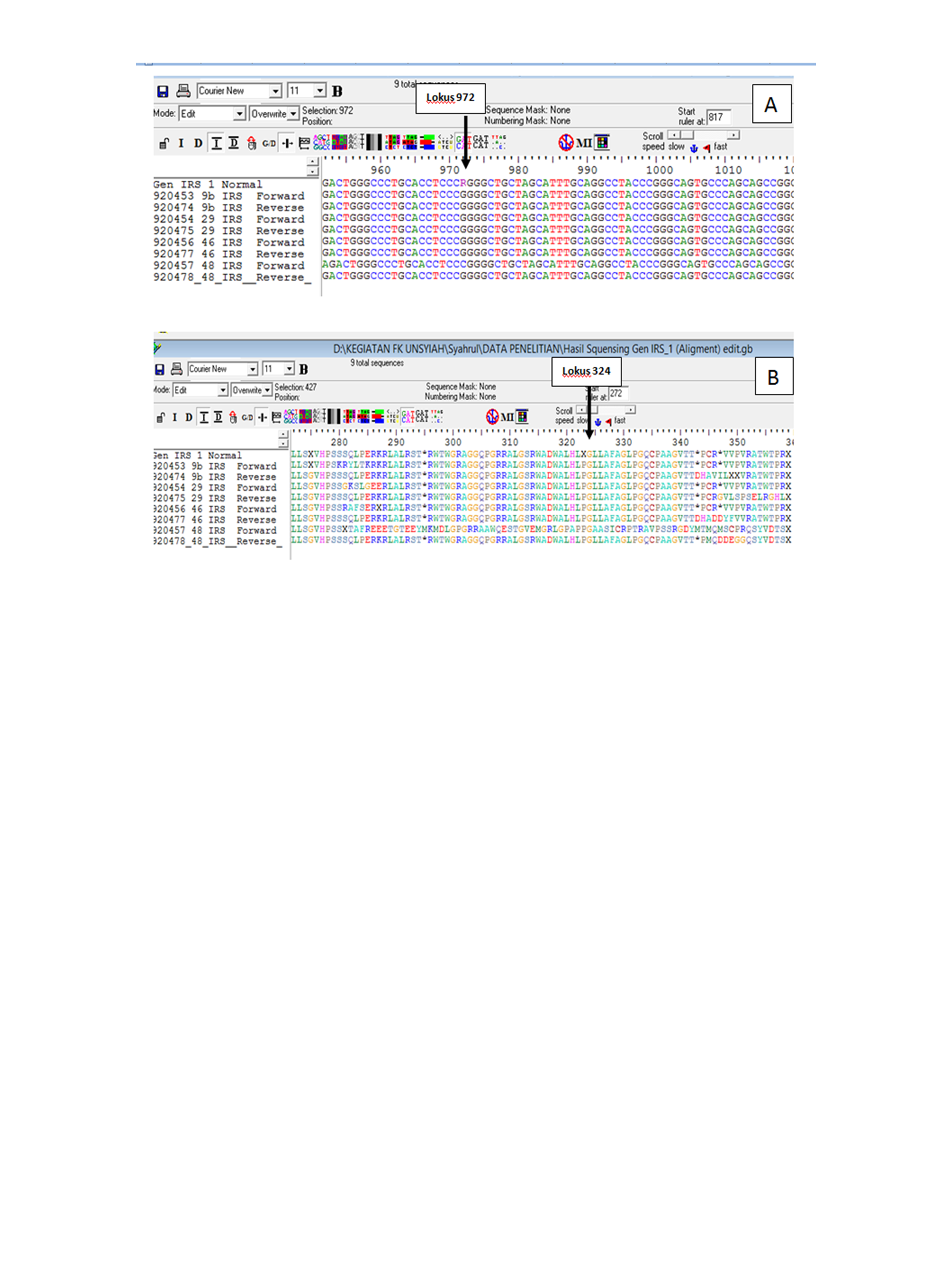

Supplement: Supplementary file 1 — Additional file 1. The result of the sequence analysis of several sample research to identify IRS-1 gene polymorphism Gly972Arg. (A) Image A shows locust 972 where mutation G → R frequently occurs. (B) Image B shows locust 324 where in the sequence of amino acid group Lysine to Arginine mutation. [file 13104_2018_3823_MOESM1_ESM.tif]
